# Soil-Transmitted Helminth Reinfection and Associated Risk Factors among School-Age Children in Chencha District, Southern Ethiopia: A Cross-Sectional Study

**DOI:** 10.1155/2016/4737891

**Published:** 2016-01-28

**Authors:** Zerihun Zerdo, Tsegaye Yohanes, Befikadu Tariku

**Affiliations:** ^1^Department of Medical Laboratory Sciences, College of Medicine and Health Sciences, Arba Minch University, 21 Arba Minch, Ethiopia; ^2^Department of Nursing, College of Medicine and Health Sciences, Arba Minch University, 21 Arba Minch, Ethiopia

## Abstract

Mass drug administration (MDA) to the most risky population including school-age children (SAC) is the central strategy to control soil-transmitted helminth (STH) infection. The present study was aimed at estimating the prevalence of STHs reinfection three months posttreatment and associated risk factors among SAC in Chencha district. A cross-sectional study design was employed from April 20 to May 5, 2015, to enroll 408 SAC. Structured questionnaire and Kato-Katz thick smear technique were used to interview parents or guardians and quantify the number of eggs per gram of stool. Pearson chi-square and logistic regression were used to assess the association between predictor variable and STH reinfection. The prevalence of STHs within three months of mass chemotherapy among SAC was 36.8% which is 93.4% of the prevalence (39.4%) before treatment. The estimated prevalence of reinfection (95%CI) for* Ascaris lumbricoides*,* Trichuris trichiura*, and hookworms was 23.8% (21.1–28.2), 16.2% (12.7–20.1), and 1.0% (0.3–2.5), respectively. Children of merchant fathers were more likely to be reinfected by STHs in Chencha district. In conclusion, there is rapid reinfection after mass chemotherapy among SAC in Chencha district. Further studies should be carried out to generate cost efficient methods that can supplement mass drug administration to accelerate the control of STHs.

## 1. Introduction

Soil-transmitted helminth (STH) infections are the major cause of public health problem in the world [1]. Their infection impairs physical development, causes malnourishment [2], decreases cognitive performances [2, 3], causes anemia [4] and school absenteeism, and decreases school performance in school-age children (SAC) [2, 5]. STHs are parasitic worms of four species of nematodes:* A. lumbricoides* (roundworm),* Trichuris trichiura* (whipworm), and* Ancylostoma duodenale* or* Necator americanus* (hookworms). People get infected by these parasites through direct penetration of the skin or ingestion of eggs developed in the contaminated soil in areas where sanitation is poor [1, 6].

The global estimate of STH infection was 438.9 million, 819.0 million, and 464.6 million people for hookworm,* A. lumbricoides*, and* Trichuris trichiura*, respectively, in 2010. The highest prevalence of STH infections and vast majority of years lived with disability attributable to STH infections occur in Asia and Africa [7]. In sub-Saharan Africa (SSA), there were 866 million people infected by STH as indicated by the World Health Organization estimate 2012: the respective number (prevalence) of people infected by hookworm,* A. lumbricoides*, and* T. trichiura* was 117 million (13.6%), 117 million (13.6%), and 100.8 million (11.6%), respectively [8].

Children at school and preschool age are a heavily affected group of the population by STHs [9]. The highest prevalence of STH infection among school-age children was observed in Asia. More than 93% of SAC in Malaysia were infected by any of the STH parasites while above two-thirds of SAC harbor one or more of the three STHs in Africa [5, 8, 10]. There were 89.9 million SAC who were infected by STHs in SSA: 117.7 (13.6%), 117.9 (13.6%), and 110.8 (11.6%) million people were infected by hookworm,* A. lumbricoides*, and* T. trichiura*, respectively [11].

One-third of the Ethiopians are infected by* A. lumbricoides*, one-quarter are infected by* T. trichiura*, and one in eight is infected by hookworms. These make Ethiopia the 2nd and the 3rd high burden country in terms of Ascariasis and hookworm infection in SSA [12]. The burden of STH among SAC in Ethiopia varies according to geographical location, type of school attended, and population [13]. The prevalence of STH infection among SAC in Chencha town [14] was 63% while it was 18% in Gondar city, northern Ethiopia [15]. In Durbete town, northwestern Ethiopia, more than half of the SAC were harboring these parasites [16].

There are different factors associated with increased transmission of STH among SAC in different parts of the world. In Ecuador, children born from mothers infected by these helminths during pregnancy had increased susceptibility to infection during early child life [17]. Male and SAC living in rural areas of Honduras where access and practice of sanitation are poor were heavily infected by STHs [18]. A systematic review and meta-analysis indicated that access and good practice of water, sanitation, and hygiene (WASH) reduce a minimum of 33% odds of infection with STHs. Treated water and soap use and availability have protective effect on any of the STH infections [19, 20]. In addition, STH infection was more common among children from low socioeconomic status. Parents who had low education status and low annual income had positive effect on the transmission of STHs among their children in Honduras [14, 21].

WHO and its partners analyzed the evidence and best practice and recommended common integrated approaches to be used for the prevention and control of STH infections and other NTDs [22]. In countries of high endemicity of STHs, preventive chemotherapy is the main strategy to control morbidity. However, rapid reinfection after successful treatment is one among the major challenges of mass drug administration. In China, after effective treatment with Albendazole, more than 83% of SAC were reinfected by* A. lumbricoides* six months posttreatment [23]. In rural Yunnan area, China, about three-fourths of school children were reinfected at the 4th month of the effective three-day 400 mg Albendazole treatment [24]. In another similar study carried out in rural aboriginal school in Malaysia, after three months of 400 mg daily of Albendazole for three consecutive days, the rate of reinfection was 45.9% after three months of treatment while it was 12.4% four months posttreatment in South Africa [25, 26].

Determining the rate of reinfection and its associated factors could help to design methods to supplement the existing strategies to interrupt the reinfection among SAC. However, there is scarce data about the rate of reinfection by STHs after mass chemotherapy among SAC in Ethiopia. Therefore, the present study was to estimate the frequency of reinfection of STH after three months of single doze Albendazole mass treatment among SAC in Chencha district, southern Ethiopia.

## 2. Methods and Materials

### 2.1. Study Area and Population

The study area is located in Southern Nations, Nationalities, and Peoples' Regional (SNNPR) state in Ethiopia. SNNPR is one of the nine regional states in Ethiopia and consists of 14 zones and 5 special districts. Gamo Gofa zone is one of the aforementioned zones and its administrative center is Arba Minch town. Chencha district where the present study was carried out is located about 40 km in the northwestern part of Arba Minch town and about 480 km in the south direction from Addis Ababa. It has 45 rural kebeles (the smallest administrative unit) and three urban and two semiurban kebeles. A total of 140,321 people were living in Chencha district. Out of these, 17,896 (12.5%) were SAC. There are 68 primary schools in the district indicating that there is more than one school in some of the kebeles. A total of 17,180 (8,892 males and 8,298 females) SAC are there in the 61 rural and 7 urban and semiurban primary schools (containing 1,661 males and 1,395 females). This revealed that 96% of the SAC were attending their education in Chencha district.

Chencha district health office in collaboration with education office of the district had administered single dose of Albendazole for all school children in the 68 primary schools within five days starting in December 2014. Children from grade one to four and of age between 5 and 14 were included in the study.

### 2.2. Study Design and Sample Size Determination

A cross-sectional study design was used to estimate the prevalence of STH reinfection within three months of single doze Albendazole treatment. The sample size was calculated by using single proportion formula based on the following assumptions: level of confidence is 95%, 50% of SAC are reinfected by any of the STHs three months after mass chemotherapy, 5% is taken as margin of error, and 10% nonresponse rate is added. Hence,

(1) $$\begin{array}{c} n={\left(\;\frac{Z\alpha }{2}\;\right)}_{\times P\left(1-P\right)/d^{2}}^{2}. \end{array}$$

Based on the above assumptions and using single population proportion formula for sample size calculation, the minimum sample size was 423.

### 2.3. Sampling Technique

Stratified sampling technique was used to select study participants from the three areas of the district: urban, semiurban, and rural areas. The number of children to be enrolled in the study from urban, semiurban, and rural areas of the district was dependent on the total number of children in each stratum. Accordingly, one urban, one semiurban, and eight rural kebeles were randomly selected to enroll students in the study. From all selected schools, students from each level of grade were selected again based on the proportion of students in each level of education. Finally, research participants from each section were selected by systematic random sampling technique by using class roster as sampling frame.

### 2.4. Method of Data Collection

After written informed assent was obtained from guardians or parents, data was collected from students and their parents (guardians) on sociodemographic factors and water access sanitation and hygiene practice of school children at both home and school environments. Pretested structured questionnaire was used for face-to-face interview with the parents and children on the sociodemographic factors. The presence of water access and sanitation at school was observed by the data collectors using checklist.

Polyethylene screw cupped stool container was given to selected students to bring stool immediately after interview. The fresh collected stool specimen was transferred to Chencha District Hospital laboratory on the same day of its collection. Kato-Katz thick smear was prepared from each specimen using a template of 41.7 mg as recommended by WHO and examined by trained laboratory technologist systematically within 30 to 60 minutes after preparation under bright field microscope. The number of helminths eggs for the three species was counted separately and the numbers obtained were multiplied by 24 in order to obtain the number of parasites per gram of stool. Egg counts were used to classify the intensity of infection into light, moderate, or heavy infections [27]. All children infected by at least one of these parasites were given 400 gm Albendazole for three consecutive days by the data collectors to ensure that they have consumed the drug.

### 2.5. Statistical Analysis

All collected information in the questionnaire and laboratory result were checked for completeness before entering the data into EpiData version 3.1. The data was exported from EpiData to STATA version 11 statistical software for analysis. Pearson chi-square was used to assess the association between independent variables considered and reinfection of STHs after mass drug administration. Logistic regression was used to compute odds ratio and corresponding 95% confidence interval for those variables significantly associated in chi-square. *P* values less than 0.05 were considered as statistically significant.

### 2.6. Ethical Consideration

The study protocol is approved by the Research Ethics Review Committee of College of Medicine and Health Sciences, Arba Minch University. Permission letter to conduct the study in the selected schools of the district is obtained from health and education office of Chencha district. Finally, informed written assent was obtained from parents or guardians of the children.

## 3. Result

### 3.1. Sociodemographic Factors and STH Reinfection

Of 423 students approached in 10 primary schools in Chencha district, 408 (96.4%) students were interviewed and submitted stool specimen for Kato-Katz thick smear preparation. There were 206 (50.5%) female students and majority of them (87%) were from rural area. The mean age of these children was 9.8 years with the standard deviation (SD) of 1.9.

Majority (62.3%) of children originate from families where there was no regular monthly income. More than three-fourths of the family's source of income was either farming or weaving and above one-quarter of the SAC's fathers were weavers who prepare Ethiopian cultural cloth. Eighty-seven percent of pupils originate from family where there was no regular income or income below 250 Ethiopian birr per a month. Except for seven, all children were living in private house with the mean household size of seven individuals (SD = 2.8). The mean age of SAC's mothers was 38.6 years (SD = 6.9); more than half were housewives and about 93% of them had no formal education or were at primary level of education. The detailed sociodemographic characteristics of the study participants were presented in Table 1.

The overall prevalence of pretreatment of STH infection was 39.4% while the reinfection with one or more of the three major groups of STH parasites within three months after treatment was 36.8% with 95% confidence interval (CI) of 32.1% to 41.6%. Of 408 school-age children involved in the study, 95 (23.2%), 66 (16.1%), and 4 (1.0%) were infected by* A. lumbricoides*,* T. trichiura*, and hookworm, respectively. The respective median egg count per gram of stool specimen for* A. lumbricoides*,* T. trichiura*, and hookworm infections was 144, 72, and 72. However, all infected children had light intensity of infection. None of the children had multiple infections with three parasites but 16 (3.9%) of the total children studied and 10.7% of the children harboring parasites were infected by both* A. lumbricoides* and* T. trichiura*. All multiple infections occurred among children living in the rural parts of the district. The prevalence of reinfection by one or more of the three STH parasites was higher among grade three students. Children in grade three were 1.2 (95% CI: crude odds ratio (COR) = 0.8–1.9; *P* = 0.350) times at increased odds of infection by any of the helminths and 1.7 (95% CI: COR = 1.02–2.70; *P* = 0.042) times more likely to be infected by* A. lumbricoides.* None of the sociodemographic factors are significantly associated with any of the STH reinfections except for children whose fathers were merchants (Table 2). Children from fathers whose occupation was merchant were at about 3 times increased odds of reinfection by any of the STHs (95% CI: COR = 1.1–8.5; *P* = 0.018).

### 3.2. WASH and STH Reinfection

There was water for children to drink and wash their hands in all primary schools in Chencha district but only 59.9% of the source of drinking water at home was from tap. However, STH reinfection did not differ due to difference in the source of drinking water for the children at the place of their residence. Almost all (99.0%) pupils had toilet at their home and 276 (68.7%) of them were private and the others were common toilets. Nearly 69% of the toilets at the students living house were local pit latrines (pit without house). Children who had no latrine for defection of feces were about 5.2 times (95% CI: COR = 0.5–50.7; *P* = 0.154) more likely to be reinfected with any of the three soil-transmitted parasites. Few of the children's families utilized their night soil as fertilizers for vegetables. The prevalence of STH reinfection among children whose families utilized night soil as fertilizer was 45.5% while it was 36.7% among children from families not using night soil as fertilizer for their vegetables with COR of 0.7 (95% CI: COR = 0.2–2.3; *P* = 0.555). Less than half (47.4%) and 37.6% of the children always wash their hands before meal and after toilet, respectively. However, neither hand washing before meal nor hand washing after toilet had significant effect (*P* = 0.818 for both) on reducing the rate of reinfection after mass drug administration for SAC.

## 4. Discussion

STH reinfection three months after mass drug administration among SAC in Chencha district was high.* A. lumbricoides* was the most rapidly reinfecting helminth followed by* T. trichiura*. The rate of reinfection has reached 93.4% of the baseline prevalence estimated by Ethiopian Public Health Institute (unpublished data). However, moderate to heavy reinfection was low, indicating that SAC targeted treatment against soil-transmitted helminthiasis is not effective in preventing the transmission of the parasite but in reducing the worm load among the infected children. Children whose fathers are merchants were more likely to be reinfected by any of the three STHs while multiple infections occurred only among pupils originating from rural parts where subsequent morbidities associated with soil-transmitted helminthiasis will be worse [2].

*A. lumbricoides* reinfection indicated in a systematic review and meta-analysis carried out by Jia and colleagues was in agreement with the finding of the present study while hookworm and* T. trichiura* reinfections estimated were significantly higher than in Chencha district [28]. The overall STH reinfection three months after mass drug treatment among SAC in Chencha district was in agreement with the rate of reinfection after six months of treatment in Chawama, Lusaka, Zambia [29]. This agreement might be associated with low prevalence of the baseline infection compensated by longer duration of follow-up for estimation of reinfection in Zambia.

The estimated reinfection in the present study was lower than the estimated reinfection rate in rural aboriginal Malaysia [26, 30] and rural Yunnan, China [24], Guizhou province in southwest China [2], Bangladesh [31], and Java, Indonesia [32]. The main reason for this difference might be the difference in the baseline prevalence of STH infection or time elapsed following mass drug administration since the rate of reinfection increases as the baseline intensity of infection increases and longer follow-up after MDA [5, 33, 34].

Unlike the above, the overall prevalence of one or more of the STHs' reinfection in South Africa [25] and southern highland of Rwanda [35] was lower than our finding but hookworm reinfection was higher. The higher rate of reinfection by hookworm in South Africa might be associated with hookworm being the most probable cause of soil-transmitted helminthiasis in South Africa, while low overall prevalence may be due to slower rate of reinfection by hookworm [11, 36]. The lower prevalence of STH reinfection among children in Rwanda may be emanated from longer duration since mass chemotherapy has been implemented and resulted in low burden of background STH infection. Reinfection due to STHs was lower among rural indigenous preschool children in Panama as compared to the present study [33]. The difference might be from lower transmission rate among preschool children than in school-aged ones.

Finally, the findings of the present study shall be interpreted in light of the following limitation of the study. The first limitation was missing data for efficacy of Albendazole at baseline survey. The other limitation was recall bias by the respondents on the factors assessed. In addition, guardians or parents of the pupils might give positive response during face-to-face interview on the water access sanitation and hygiene related factors that can dilute the strength of their association with STHs reinfection.

## 5. Conclusion

There is rapid reinfection of STHs three months after mass drug administration among school-age children in primary schools in Chencha district. The prevalence of STH reinfection has reached 93.4% of the baseline prevalence among SAC in the district. This implies that mass chemotherapy targeted on SAC is not effective in preventing the transmission of the parasites. Therefore, further studies to generate cost-efficient methods to supplement the use of mass drug administration to accelerate control of soil-transmitted helminthiasis and subsequent morbidities among SAC should be done in Ethiopia and other STH infection highly endemic countries.

## Figures and Tables

**Table 1:** Sociodemographic characteristics of school-age children and STH infection among SAC Chencha district, southern Ethiopia.

Variable	Variable value	Frequency (%)	STH infected (%)	AL infected (%)	TT infected (%)
Place of residence	Rural	359 (87.8)	131 (36.8)	85 (23.8)	59 (16.5)
	Semiurban	21 (5.1)	5 (23.8)	3 (14.3)	2 (9.5)
	Urban	29 (7.1)	13 (44.8)	8 (27.6)	5 (17.2)

Sex of SAC	Male	203 (49.5)	76 (37.62)	51 (25.2)	29 (14.4)
	Female	207 (50.5)	74 (35.92)	46 (22.3)	37 (18.0)

Age of SAC	5–9	174 (42.4)	72 (41.4)	44 (25.3)	37 (21.3)
	10–14	236 (57.6)	78 (33.3)	53 (22.7)	29 (12.4)

Level of education (grade)	One	66 (16.1)	29 (43.9)	19 (28.8)	13 (19.7)
	Two	99 (24.2)	33 (33.3)	19 (19.2)	13 (13.1)
	Three	114 (27.8)	46 (40.4)	35 (30.7)	20 (17.5)
	Four	131 (31.9)	42 (32.6)	24 (18.6)	20 (15.5)

House ownership	Owned	402 (98.3)	146 (36.5)	96 (24.0)	62 (15.5)
	Rented	7 (1.7)	3 (42.9)	1 (14.3)	3 (42.9)

Household size	< or = 5	95 (24.2)	27 (28.4)	16 (16.8)	15 (15.8)
	>5	297 (75.8)	112 (38.0)	73 (24.8)	47 (15.9)

Age of child's mother	< or = 35	149 (37.5)	54 (36.5)	35 (23.7)	24 (16.2)
	36–45	192 (48.4)	70 (36.7)	46 (24.1)	30 (15.7)
	>45	56 (14.1)	23 (41.1)	14 (25)	11 (19.6)

Mother's educational status	< or = primary	372 (93.0)	136 (36.8)	85 (22.9)	61 (16.5)
	5–10	19 (4.75)	7 (36.8)	7 (36.8)	3 (15.8)
	>10	9 (2.25)	3 (33.3)	2 (22.2)	1 (11.1)

Occupation of mothers	Housewife	206 (51.2)	74 (36.3)	52 (25.5)	28 (13.7)
	Merchant	46 (11.4)	22 (47.8)	12 (26.1)	12 (26.1)
	Civil servant	11 (2.7)	4 (36.4)	2 (18.2)	2 (18.2)
	Farmer	127 (31.6)	42 (33.1)	25 (19.7)	20 (17.8)
	Daily laborer	12 (3.0)	5 (41.7)	4 (33.3)	3 (25.0)

Occupation of child's father	Weaver	114 (27.9)	35 (31.0)	26 (23.0)	12 (10.6)
	Merchant	16 (3.9)	10 (62.5)	5 (31.2)	5 (31.3)
	Civil servant	31 (7.6)	12 (38.7)	7 (22.6)	6 (19.4)
	Farmer	209 (51.1)	75 (36.1)	48 (23.1)	33 (15.9)
	Others	39 (9.5)	17 (43.6)	10 (25.6)	10 (25.6)

AL: *A. lumbricoides*; TT: *T. trichiura*.

**Table 2:** Univariate logistic regression of school-age children's sociodemographic factors and any of the three STH infections in Chencha district, southern Ethiopia.

Variable	Variable value	Frequency (%)	STH infected (%)	Crude OR (95% CI)	*P* value
Place of residence	Rural	359 (87.8)	131 (36.8)	1	
	Semiurban	21 (5.1)	5 (23.8)	0.5 (0.19–1.51)	0.238
	Urban	29 (7.1)	13 (44.8)	1.4 (0.65–3.01)	0.386

Sex of SAC	Male	203 (49.5)	76 (37.62)	1	0.722
	Female	207 (50.5)	74 (35.92)	0.9 (0.62–1.39)	

Age of SAC	5–9	174 (42.4)	72 (41.4)	1	0.096
	10–14	236 (57.6)	78 (33.3)	0.7 (0.47–1.06)	

Level of education (grade)	One	66 (16.1)	29 (43.9)	1	
	Two	99 (24.2)	33 (33.3)	0.6 (0.34–1.21)	0.169
	Three	114 (27.8)	46 (40.4)	0.8 (0.47–1.59)	0.638
	Four	131 (31.9)	42 (32.6)	0.6 (0.33–1.33)	0.119

House ownership	Owned	402 (98.3)	146 (36.5)	1	0.730
	Rented	7 (1.7)	3 (42.9)	1.3 (0.29–5.91)	

Household size	< or = 5	95 (24.2)	27 (28.4)	1	0.092
	>5	297 (75.8)	112 (38.0)	1.5 (0.93–2.55)	

Age of child's mother	< or = 35	149 (37.5)	54 (36.5)	1	
	36–45	192 (48.4)	70 (36.7)	1.0 (0.64–1.57)	0.975
	>45	56 (14.1)	23 (41.1)	1.2 (0.65–2.27)	0.547

Mother's educational status	< or = primary	372 (93.0)	136 (36.8)	1	
	5–10	19 (4.75)	7 (36.8)	1.0 (0.39–2.61)	0.994
	>10	9 (2.25)	3 (33.3)	0.9 (0.21–3.49)	0.833

Occupation of mothers	Housewife	206 (51.2)	74 (36.3)	1	
	Merchant	46 (11.4)	22 (47.8)	1.6 (0.84–3.07)	0.148
	Civil servant	11 (2.7)	4 (36.4)	1.0 (0.28–3.54)	0.995
	Farmer	127 (31.6)	42 (33.1)	0.9 (0.54–1.38)	0.553
	Daily laborer	12 (3.0)	5 (41.7)	1.2 (0.38–4.09)	0.707

Occupation of child's father	Weaver	114 (27.9)	35 (31.0)	1	
	Merchant	16 (3.9)	10 (62.5)	3.7 (1.25–11.02)	0.018
	Civil servant	31 (7.6)	12 (38.7)	1.4 (0.62–3.21)	0.417
	Farmer	209 (51.1)	75 (36.1)	1.3 (0.77–2.05)	0.360
	Others	39 (9.5)	17 (43.6)	1.7 (0.82–3.64)	0.154
